# Study on the zona pellucida 4 (ZP4) gene sequence and its expression in the ovaries of patients with polycystic ovary syndrome

**DOI:** 10.1007/s40618-015-0260-4

**Published:** 2015-03-05

**Authors:** B. Meczekalski, R. Nawrot, W. Nowak, A. Czyzyk, H. Kedzia, A. Gozdzicka-Jozefiak

**Affiliations:** 1 Department of Gynecological Endocrinology, Poznan University of Medical Sciences, Polna 33, 60-535 Poznan, Poland; 2 Department of Molecular Virology, Faculty of Biology, Adam Mickiewicz University in Poznan, Umultowska 89, 61-614 Poznan, Poland; 3 Department of Molecular Biology Techniques, Faculty of Biology, Adam Mickiewicz University in Poznan, Umultowska 89, 61-614 Poznan, Poland; 4 Pathomorphology Unit, Gynecological-Obstetrical Hospital in Poznan, Polna33, 60-535 Poznan, Poland

**Keywords:** Polycystic ovary syndrome, Zona pellucida, ZP4, Oocyte-specific genes

## Abstract

**Background:**

Polycystic ovary syndrome (PCOS) is a common endocrine disorder of unknown pathology, involving reproductive and metabolic abnormalities. Oocyte-specific genes are a group of genes expressed exclusively in ovarian tissue; therefore, they can play an important role in ovarian pathologies such as PCOS. The zona pellucida 4 (ZP4) gene encodes glycoprotein which is a part of the extracellular matrix of oocyte.

**Materials and methods:**

We analyzed 87 patients with PCOS, which were divided into four groups depending on their phenotype. In each patient, we performed profound clinical and biochemical analysis, including the measurement of serum androgens. The ovarian tissue samples were used to perform a real-time polymerase chain reaction and immunohistochemical staining using anti-ZP4 monoclonal antibodies. The ZP4 gene was sequenced from peripheral lymphocytes.

**Results:**

The expression of ZP4 was present in early antral follicles and was stronger in mature follicles. The subgroup of patients with eumenorrhea and without hyperandrogenism presented the highest expression of ZP4 in ovarian tissue. In one case, we found a mutation of the ZP4 gene. No correlations were found between the ZP4 expression level and biochemical or clinical indices.

**Conclusions:**

Data from this and animal studies suggest a possible relationship between androgens and ZP4 expression. ZP4 expression is highest among patients with PCOS and a regular cycle, and this is a consequence of the presence of mature follicles in this group. In some patients with PCOS and infertility, ZP4 mutation can be found.

## Introduction

Polycystic ovary syndrome (PCOS) is one of the most common endocrinopathies, affecting 5–10 % of women of reproductive age [1]. The syndrome is a complex phenomenon, which involves the reproductive and other systems. Most predominant and concomitantly diagnostic features of the syndrome include ovulation disorders (oligo- or anovulation), polyfollicular structure of the ovary and hyperandrogenemia along with its clinical features, i.e., hirsutism, acne and androgenic alopecia. The clinical manifestations of ovulation disturbances are menstrual disturbances (oligomenorrhea and amenorrhea), which are related to decreased fertility. In addition, metabolic disturbances are common in this syndrome. The incidence of obesity, insulin resistance, glucose intolerance and dyslipidemia is increased among patients with PCOS [1]. Many factors are believed to play a role in the pathogenesis of PCOS. These include environmental, lifestyle and genetic factors [2, 3].

To date, more than 70 genes have been studied in order to elucidate whether they play a role in the pathogenesis of PCOS. The genes involved in diverse processes that have been studied range from those engaged in steroidic hormones synthesis, oocyte development, as well as the genes of hormone receptors, through to those important for glucose metabolism, insulin synthesis or action, and energetic homeostasis [2, 3]. Recent studies have shown that one important pathophysiologic feature of PCOS is an impairment of follicular development and the development of oocytes. So far, it is known that the transforming growth factor-beta family signaling pathway is disturbed in the oocytes of women with PCOS. Some members of this protein family are expressed exclusively in oocyte, and that is why they are referred to as oocyte-specific genes. Disturbances concerning the function of oocyte-specific genes are thought to play an important role in the pathology of some diseases of the reproductive system. They may be implicated in infertility, premature ovarian failure and PCOS [4].

The zona pellucida (ZP) genes are a group of oocyte-specific genes. ZP is an extracellular matrix that surrounds growing oocytes, ovulated eggs and preimplantation embryos. These genes play an important role during sperm–egg interaction but also in folliculogenesis. ZP4 glycoprotein was the last ZP protein identified in humans, and its exact role is still not fully recognized [5]. All the zona proteins possess the archetypal “ZP domain.” This “ZP domain” consists of approximately 260 amino acids, including eight conserved Cys residues, and is predicted to have high *β*-strand content with additional conservation of hydrophobicity, polarity and turn forming tendency at a number of positions [14]. The human Zp4 gene (pseudogene in the mouse) of 8226 bp, located on chromosome 1 (1q43), encodes a 540 amino acids long polypeptide. The ZP4 gene covers 12 exons and 11 introns. The open-reading frame contains 1623 bp [6]. On the basis of the ZP4 expression pattern in the ovary of the rat, it is suggested that this gene plays an important role in primordial follicle formation [unpublished data]. Structurally, human ZP4 protein is most similar to human ZP1 and that is why it was previously misidentified as ZP1. It can be supposed that ZP4 also can play a role to ensure the integrity of human ZP. What is more, studies in humans have proved that ZP4 take a part in acrosomal reaction [7–9]. ZP4 induces acrosomal exocytosis through a Gi-independent pathway [8]. Binding sites for recombinant ZP4 glycoprotein are located both at the N- and C-terminus of proacrosin [10].

Regarding these findings and previous studies of other oocyte-specific genes, the aim of this study was to analyze the ZP4 coding sequence and its expression in patients with polycystic ovary syndrome. We also decided to investigate the localization of ZP4 protein in the ovaries of patients with PCOS.

## Materials

In the study, 87 patients (mean age 24.7 ± 3.91 years; mean BMI 24.7 ± 5.2 kg/m^2^) with PCOS were included. The PCOS was diagnosed according to the European Society of Human Reproduction and Embryology and American Society of Reproductive Medicine (ESHRE/ASRM) criteria from 2003, namely: oligo- and/or anovulation (OM), clinical and/or biochemical signs of hyperandrogenism (HA), or polycystic ovaries in ultrasound (US), after exclusion of other known etiologies. For establishing the diagnosis, two out of three criteria must be fulfilled; therefore, four different phenotypes of PCOS arise according to the combination of its three main manifestations: 1: OM + HA + US, 2: HA + US, 3: OM + US and 4: OM + HA (Table 4).

A total testosterone serum level above 0.8 ng/ml was considered as hyperandrogenemia. Patients with a history of the following conditions and procedures were excluded: ovarian surgery, radio- or chemotherapy, premature ovarian failure, hyperprolactinemia, thyroid dysfunction or ovulation induction in the last 3 months.

All the studied subjects gave their written consent to participate in the study. The Ethical Commission of Poznan University of Medical Sciences approved the study’s protocol.

## Methods

In each case, we took detailed medical history, with special emphasis on: the regularity of menses and the age of menarche. To assess the phenotype and signs of hyperandrogenism, physical and gynecological examination has been performed; the hirsutism was quantified on the basis of modified Ferriman–Gallwey scale by the same physician in each case. To confirm the PCOS diagnosis and exclude other possible causes of presented clinical picture, we performed hormonal analysis, which has been made in eumenorrheic and oligomenorrheic women in late follicular phase (from 10th to 12th day of menstrual period) or in any day in amenorrheic patients. Following hormones, concentrations have been measured in serum: estradiol (E2), follicle-stimulating hormone (FSH), luteinizing hormone (LH), testosterone (T), dehydroepiandrosterone sulfate (DHEAS), 17-OH progesterone (17-OHP).

Serum levels of insulin were determined by ELISA (Enzymun Test Insulin; Boehringer Mannheim, Mannheim, Germany). T, LH, FSH and PRL were measured with specific chemiluminescence assays (Chiron Diagnostics GmbH, Fernwald, Germany).

The following were measured with specific RIAs: sex hormone-binding globulin (SHBG) (Orion Diagnostica, Espoo, Finland), DHEAS (DPC, Los Angeles, CA), and 17a-hydroxyprogesterone and IGF-I (Biosource Europe S.A., Nivelles, Belgium). Sampled sera were stored at 220 °C until analysis was performed. The free T index and the ratio of fasting glucose to insulin were calculated. Plasma glucose was determined instantaneously with a chemiluminescence assay (Chiron Diagnostics GmbH). The intraassay and interassay coefficients of variation were 10 % for all assays performed.

The blood samples were taken in the morning after at least 8 h of fasting and stored at −20 °C for DNA isolation.

### Ovarian specimens

Ovarian samples were obtained from 87 women undergoing ovarian wedge resection because of PCOS. All the women were recruited from among patients of the Department of Gynecological Endocrinology of Poznan University of Medical Sciences. The ethnicity of all subjects was Caucasian, and they were unrelated to one another.

The ovary samples were cut into uniform-size slices and frozen in −70 °C for further studies.

### DNA isolation and PCR amplification

In the women with PCOS, we performed ZP4 gene sequencing. The DNA was extracted from blood cells using the QIAamp DNA Blood Mini Kit (Qiagen). Genomic DNA was used for in vitro amplification by PCR with a specific set of primers complementary to the coding sequence of the ZP4 gene (Table 1).

**Table 1:** Primers used for PCR study of ZP4 gene sequence

Primer name	Sequence (5′ → 3′)	Product length (bp)
ZP4-1f	AGGAATCTGGGCAGGCAGAC	947
ZP4-2r	CAGGTGTCACTGCACAGAGCA	
ZP4-3f	ATAGCAGGGCACTCTTACAAGG	527
ZP4-4r	AGAAGTCTGTTACATTGGGATCAAG	
ZP4-5f	GTTGTCACTGGGAAGTTGTCAC	1076
ZP4-6r	ACAGATGGACCTAATCAAGTAATGG	
ZP4-7f	GCACTTAATGCTTTCCTTGAGTTAA	695
ZP4-8r	GGGTCTGATAGTTGTCTCCAATGTA	
ZP4-9f	GTGGTTCTTTCATACACCTGTGCT	1076
ZP4-10r	GCCAGAGACTAGGAAAGGTTAGACA	
ZP4-11f	ACATCTTTATACGAAAGTCCTGGTG	695
ZP4-12r	TTTATTCCACGTTCTCTGACCTAAG	

PCR was performed in the UNO II thermocycler (Biometra, Germany). Initial DNA denaturation was performed at 95 °C for 5 min, then step 2—denaturation at 95 °C for 20 s—followed, ensued by primers annealing at 56 °C for 15 s and elongation at 72 °C for 80 s. The cycle of denaturation, annealing and elongation was repeated 35 times and followed by final elongation at 72 °C for 5 min. The purified PCR products were ligated into a pGEM-T Easy vector (Promega). Competent *Escherichia coli* DH5alpha cells were transformed with ligation products. Plasmids with inserts were extracted from the white-transformed colonies with a QIAprep Plasmid Kit (Qiagen). Inserts and PCR products were sequenced with an automated 3130 × Genetic Analyzer (Applied Biosystems) in the Faculty of Biology, Adam Mickiewicz University in Poznan. The sequences that were obtained were analyzed and compared to those available in the GenBank database (NCBI, USA) using BLAST search.

### Real-time PCR

The ovarian tissue samples were quickly cut into 30-mg samples, placed in 1.5-ml tubes and stored in the RNA stabilization reagent (Qiagen) at −80 °C until further processing. Disruption and homogenization of the tissue samples were carried out with a mortar and pestle.

Total RNA was isolated using the RNeasy Mini Kit (Qiagen) according to the manufacturer’s instructions. After isolation, the RNA was stored at −80 °C.

Reverse transcription and real-time PCR were performed with a designed set of primers (Table 2) for the human ZP4 (zona pellucida 4) coding sequence, glyceraldehyde-3-phosphate dehydrogenase (GAPDH), human R2A polymerase (polR2a) and human hypoxanthine phosphoribosyltransferase 1 (HPRT). The primers were designed to cross exon–intron boundaries. The possibility of DNA co-amplification was excluded from failure of the product detection in real-time PCR with the DNA template.

**Table 2:** Primers used for real-time PCR analyses

Primer name	Sequence (5′ → 3′)	Product length (bp)
ZP4-15f	GCGGCTGAACACAAGGTGGT	107
ZP4-16r	CGTGCTGGGATGGAGTCACA	
PolR2a-1f	GCAAATTCACCAAGAGAGAC	163
PolR2a-2r	ATGTGACCAGGTATGATGAG	
HPRT1-1f	TGGTCAGGCAGTATAATCCAAAGA	100
HPRT1-2r	TCAAATCCAACAAAGTCTGGCTTA	
GAPDH_A	TTCGTCATGGGTGTGAACC	106
GAPDH_B	GATGATGTTCTGGAGAGCCC	

Reverse transcription reactions were conducted using the QuantiTect Reverse Transcription Kit (Qiagen) according to the manufacturer’s instructions.

The expression level of each gene was measured by means of real-time PCR experiments using the QuantiFast SYBR Green PCR Kit (Qiagen). Quantification of mRNA samples was carried out by relating the PCR threshold cycle obtained from tissue samples to specific standard curves. The relative abundance of the target was divided by the relative abundance of GAPDH, PolR2a and HPRT in each sample to generate a normalized abundance (NF–normalization factor).

### Immunohistochemistry

Material for immunohistochemical analysis included cut fragments of ovarian tissue received from patients with polycystic ovary syndrome. Fragments of the ovarian tissue were investigated using immunohistochemical methods, which included staining using monoclonal antibodies from mice (MA-1671 clone) against the human ZP4 antigen, hematoxylin–eosin staining, immunohistochemical staining for the presence of alpha-inhibin and PAS reaction for the presence of polysaccharides in the tissue. Immunoreaction was conducted with the EnVision™^+^ System HRP.

### Statistical analysis

Results are presented as means and standard deviation [SD]. Statistical analysis was performed using the software StatSoft 2011 STATISTICA version 10. The normality of data distribution was verified with the Shapiro–Wilk test. The variables were verified by tests: the parametric Student’s* t* test or nonparametric Mann–Whitney* U* test. A *p* value ≤0.05 was considered significant. Interactions between the variables were tested using Pearson’s linear correlation analysis.

## Results

### ZP4 sequence analysis in blood samples

From a total of 87 patient blood samples with PCOS, we identified four nucleotide changes in the ZP4 coding sequence: three silent nucleotide changes in exons 1, 4, 10 and one nucleotide change in the exon 5 (position 114, T > G) (Table 3). The mutation in exon 5 (T > G) resulted in substitution of cysteine for glycine of the amino acid in position 223 of the ZP4 protein. Cysteine in this position is strictly conserved in ZP4 protein. The point mutation in one of the ZP4 alleles could contribute to the expression of two ZP4 protein forms—one protein with normal function and one incorrectly folded, with disturbed function of the zona pellucida domain.

**Table 3:** Nucleotide changes in the ZP4 coding sequence from a total of 87 patient samples with PCOS and resulting amino acid changes in the ZP4 protein

Sample no.	Exon number of ZP4 gene	Nucleotide change, (position in exon)	Amino acid change in ZP4 protein, (position in protein, gi 10863987)
2	5	T → G, position 114	C → G, position 223
9	4	T → A, position 68	No change
12	10	C → T, position 75	No change
13	1	C → T, position 18	No change

All changes are monoallelic

### Ovarian samples: characteristics of women diagnosed with PCOS

Because of ethical considerations, we could not collect a control group for expression studies. All the surgical procedures performed on gonads bring a risk of reducing the follicular reserve, which is ethically unacceptable in healthy women. Instead, we decided to compare the ZP4 expression level between women with different phenotypes of PCOS. The phenotypes were divided according to the Rotterdam criteria [1].

Of the 87 PCOS subjects studied, the largest phenotype group included women with all three features, namely OM + HA + US (38 %, Table 4). Another 16 % had HA + US (the ovulatory phenotype), and 14 % had OM + US (the nonhyperandrogenic phenotype).

**Table 4:** Clinical and biochemical characteristics of studied groups

Characteristics (mean ± SD)	First phenotype: OM + HA + US	Second phenotype: HA + US	Third phenotype: OM + US	Fourth phenotype: OM + HA
Age (years)	25.2 ± 5.1	24.1 ± 6.3	25.2 ± 4.8	24.9 ± 5.2
BMI (kg/m^2^)	24.6 ± 6.2	25.7 ± 6.0	24.6 ± 4.6	24.9 ± 4.9
FSH (mIU/ml)	5.94 ± 1.40	6.61 ± 1.62	6.08 ± 1.89	6.20 ± 1.83
LH (mIU/ml)	10.89 ± 3.52*	14.09 ± 5.26	14.13 ± 6.73*	14.12 ± 6.38*
E2 (pg/ml)	68.35 ± 42.77	62.19 ± 30.15	58.75 ± 29.13	59.52 ± 29.16
T (ng/ml)	0.70 ± 0.29	0.89 ± 0.39	0.78 ± 0.30	0.81 ± 0.33
SHBG (mIU/ml)	51.37 ± 24.52	48.00 ± 24.52	44.93 ± 46.62	45.65 ± 42.32
DHEAS (ng/ml)	3.52 ± 2.73	2.65 ± 1.63	2.89 ± 1.41	2.83 ± 1.45
Insulin (mIU/ml)	10.05 ± 6.92	12.25 ± 10.16	9.19 ± 5.81	9.87 ± 7.04
PRL (ng/ml)	20.47 ± 8.87	18.00 ± 7.50	20.59 ± 14.64	19.97 ± 13.30

The statistically significant differences between studied and control group are marked: (* OM + HA + US vs OM + US and OM + HA)

Interestingly, 24 % did not meet the criteria for PCO-US; these subjects were separately analyzed in the separate group OM + HA. The clinical and biochemical characteristics of all the studied groups are shown in Table 4.

### Real-time PCR results

The results of the study show that the expression level of ZP4 gene in the ovaries of women with PCOS was very low comparing to other genes with a constitutive expression. The mean number of copies for the GAPDH constitutive gene was 39,287, for the PolR2a constitutive gene 41,854 and for HPRT 22,035.

**Fig. 1:**
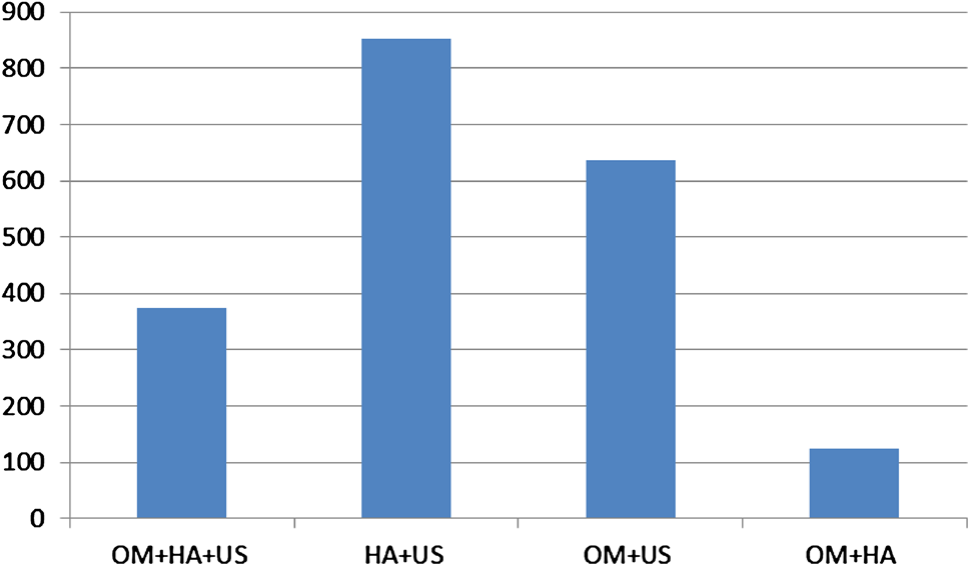
Mean ZP4 expression level in studied groups (vertical—number of copies)

In the OM + HA + US group, the mean Zp4 expression level was estimated as 373.212 ± 341.75 number of copies, whereas in the HA + US group, it was 852.0 ± 742.0, in the OM + US group, it was 636.7 ± 516.51 and in the OM + HA group, it was 124.1 ± 137.8. Considering all the groups with polycystic ovaries (I + II + III) together, the mean expression level in the RT-PCR was 620.6 ± 3780.5 numbers of copies (Fig. 1). The statistical analysis revealed that the differences between all the groups were significant *p* < 0.0001 (ANOVA test). Post hoc analyses revealed that the difference was significant between groups OM + HA + US and HA + US; HA + US and OM + HA; and OM + US and OM + HA.

### Correlation analysis

We analyzed the possible correlations between all the measured hormones as well as other laboratory data (including fasting glucose levels and SHBG), but we found no significant correlations between any of those factors and the ZP4 expression level. Moreover, we checked also possible links between the clinical data: age, BMI, Ferriman–Gallwey score and ZP4 expression, but we did not find any significant connections here either.

#### Immunohistochemical analysis ZP4 protein localization

**Fig. 2:**
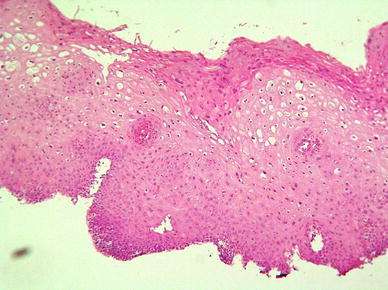
Primary ovarian follicles—no immunohistochemical reaction

**Fig. 3:**
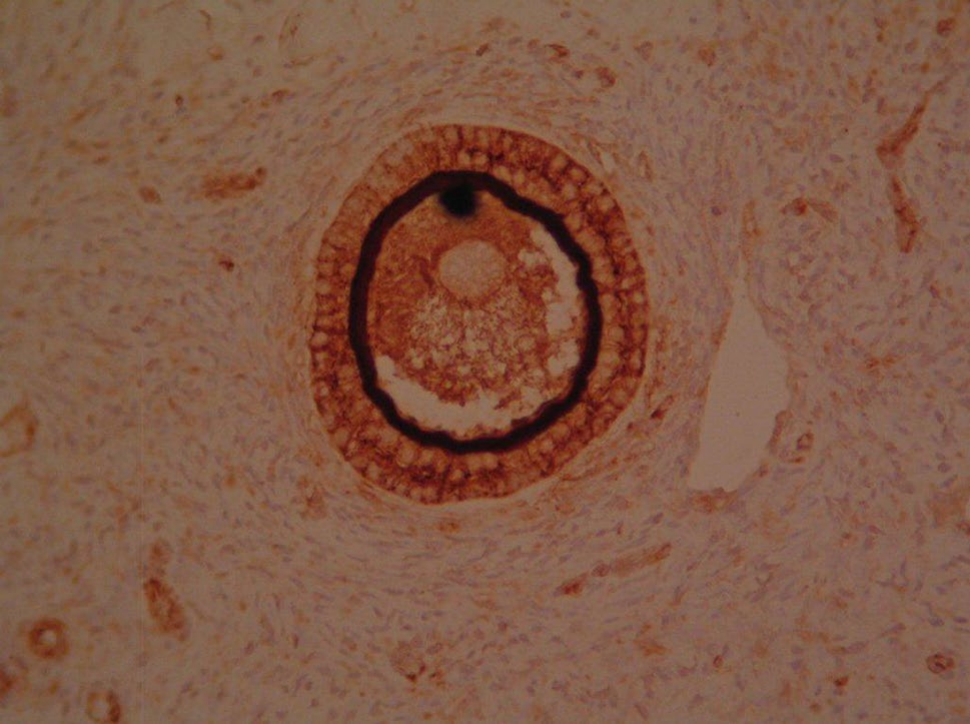
Very intense immunohistochemical reaction in follicle with antrum folliculi

As we expected, ZP4 protein was localized only in the ovarian follicles. There was no immunohistochemical reaction in the primary ovarian follicles (Fig. 2), very low reaction in single epithelial cells of secondary ovarian follicles, but very intense reaction was observed in follicles with antrum folliculi. The reaction intensity was highest in the close proximity layer of the follicle, which is corresponding to developing a zona pellucida envelope (Fig. 3). It is also suggested from the positive PAS reaction that the ZP4 antigen is polysaccharide.

## Discussion

The zona pellucida, an acellular glycoprotein matrix surrounding mammalian eggs and early embryos, mediates sperm–egg interaction, provides a postfertilization block to polyspermy and protects the embryo prior to implantation. The zona pellucida 4 gene (ZP4) is a recently identified gene from the oocyte-specific gene family. ZP4 has been purified by the immunoaffinity column and was identified as a 65 kDa protein glycolyzed mainly by N-linked carbohydrate moieties [11]. The main function of the ZP4 seems to be the induction of the acrosome reaction and the inhibition of the binding of spermatozoa to zona pellucida, a function which is shared also by the ZP3 glycoprotein. N-Glycolyzation of the protein, along with extracellular calcium concentration, is required for the action of ZP4. The function is also facilitated by signaling through the protein kinase C (PRKCA), kinase A (PRKAR1A), protein tyrosine kinase (JAK1), and the L-type and T-type calcium channels. G proteins also participated in ZP3-induced, but not ZP4-induced, acrosomal reaction [11].

In the presented study, the expression of Zp4 in polycystic ovaries is not compared to healthy controls. Because of ethical considerations, we could not collect a sufficient number of controls for this study. Obtaining functioning ovarian tissue with follicles from healthy women or those undergoing surgery for benign lesions has not been accepted by ethical committee, since those procedures are related to decrease in ovarian reserve. Instead, we compared the different phenotypes of PCOS. There is a lack of human data indicating the stage of follicle development and ZP4 expression level. The experiments performed on animal models indicate the scant expression of ZP proteins in the early stages of development, which is in line with our finding [3–5].

The immunohistochemical analysis revealed the presence of ZP4 in antral follicles and failed to find a significant reaction in the primordial follicles. The strongest reaction was present in a layer adjacent to the antral follicle which corresponds to the uniform coat that is formed around the growing oocyte [3–5].

In our study, we analyzed the ZP4 expression on mRNA level in ovarian samples taken during the wedge resection of the ovaries of patients diagnosed with different phenotypes of PCOS. All studied patients have poor response to the pharmacological treatment undergoing surgery as second-line therapy. In this syndrome, ovarian follicles are present in large numbers, but they are arrested at an early to mid (early antral follicles)-developmental state and fail to mature. Real-time PCR revealed the highest mRNA expression of ZP4 in the group of women with eumenorrhea (HA + US), and it was comparable to the expression in the OM + US group (no statistical difference was present between these groups). ZP4 expression was considerably lower in the OM + HA + US phenotype and the OM + HA. Keeping in mind the fact that immunohistochemical staining showed the highest expression of ZP4 in the mature antral follicles, and the highest number of its copies in the ovaries of women with eumenorrhea is understandable. Only in this group of women were follicles able to undergo maturation and express zona pellucida genes, whereas in patients with oligomenorrhea, the follicles are arrested in the early stages of their development [2]. In these stages, the immunohistochemistry showed a very low expression. Interestingly, also patients without hyperandrogenemia had a similar intensity of expression level. From animal studies, it is known that androgens regulate the zona pellucida genes expression, so it is possible that the lack of the excess of testosterone positively influenced the ZP4 transcription [12, 13]. The lowest levels of expression were present in the groups with oligomenorrhea and hyperandrogenemia, which partially is in line with the above hypothesis. On the other hand, in the correlation analysis, we failed to find any direct dependence between the ZP4 expression level and testosterone, the free testosterone index, the DHEAS or the Ferriman–Gallwey score. Considering the cited animal data and our data from real-time PCR in PCOS, analysis of the relation between androgens and zona pellucida genes in humans may have a big scientific potential [12, 13].

Another important part of our study was the ZP4 sequence analysis in the blood samples from PCOS patients. It showed that ZP4 nucleotide changes are present in some patients with PCOS. In one case, mutation in position 114, in exon 5, was revealed (Table 3). This mutation results in the substitution of cysteine for the glycine of amino acid in position 223 of the ZP4 protein. Cysteine in this position is strictly conserved in the ZP4 protein. This mutation is located in “ZP domain” module of ZP4 (corresponds from 188 to 460 aa). ZP domain has been shown to play an important role in the polymerization of extracellular matrix proteins, including ZP matrix [14]. Whether such mutation affects structure and function of ZP4 remains to be determined. Zona pellucida proteins are responsible for sperm–oocyte reaction, and recently, it was shown that an abnormal ZP1 gene could be responsible for familial infertility [9, 15]. The presence of ZP4 gene mutation in one case of PCOS is not sufficient to judge about its role in the pathogenesis of PCOS or infertility. However, taking into account the data from literature and our results, this fact indicates we believe the necessity of further research in this field.
